# A Transferable Evaluation Framework for Mobile Health Data Collection in Shift-Work Nurses: Development and Feasibility Study

**DOI:** 10.2196/103689

**Published:** 2026-09-30

**Authors:** Kyulhee Park, Kihye Han

**Affiliations:** 1 Department of Nursing, Chung-Ang University, 84 Heukseok-ro, Dongjak-gu, Seoul, 06974, Republic of Korea, 82 820-5995

**Keywords:** mHealth, mobile app, diary study, shift work, nurses, usability testing, feasibility study, occupational health

## Abstract

**Background:**

Shift-work nurses experience substantial variability in work schedules, health behaviors, and recovery patterns, which traditional retrospective surveys fail to capture due to recall bias. Although mobile health (mHealth) tools offer ecological momentary data capture, existing off-the-shelf survey platforms lack shift-synchronized notification logic and impose excessive cognitive friction on fatigued clinicians, limiting their applicability in nursing research.

**Objective:**

This study aimed to propose and demonstrate a transferable, dual-perspective evaluation framework for mHealth data collection tools in high-burden occupational settings, using the newly developed “Nurses’ Work-Life and Health” app as a tailored exemplar.

**Methods:**

A 3-phase, user-centered iterative design was used: (1) needs assessment, (2) app development with alpha testing (n=5) and beta testing (n=16), and (3) a 14-day feasibility and process evaluation. The feasibility study evaluated 5 shift-work nurses who completed daily near–real-time journal entries over 14 consecutive days, capturing shift characteristics, sleep, nutrition/hydration, physical activity, and acute fatigue/stress. To complement end-user evaluation (n=5), an expert nurse researcher participated in a follow-up semistructured interview assessing methodological rigor. Evaluation was guided by the technology acceptance model and system usability scale. Objective system logs and subjective surveys were integrated to analyze adherence, completion, and user burden.

**Results:**

Phase 1 needs assessment identified key functional requirements, including shift-synchronized notifications, low cognitive burden timeline entries for postshift fatigue, and automated time-stamping to verify contemporaneous logging. In phase 2, the app demonstrated high usability and acceptance, with total mean scores of 3.47 (SD 0.53) in alpha testing and 3.48 (SD 0.55) in beta testing (range 1‐4). In the phase 3 feasibility study, the app demonstrated 100% retention and 94.3% adherence (mean 13.2, SD 1.1 d). The data entry completion rate was 88.5%, with an average entry time of 3.4 minutes. Participants reported high overall satisfaction and low user burden, reflected by mean scores of 3.82 and 3.65, respectively. Time-stamped logs supported the feasibility of near–real-time data capture without disrupting clinical workflows. Qualitative feedback from the expert nurse researcher highlighted the app’s methodological strengths, including its potential to reduce recall bias and support for self-monitoring, while also identifying areas for refinement.

**Conclusions:**

By integrating subjective end-user perceptions, objective usage logs, and qualitative scrutiny from an expert researcher, this study established a transferable evaluation framework for mHealth research instruments. The findings indicate that shift-tailored mobile platforms can achieve high temporal fidelity data capture in complex clinical settings, providing a replicable evaluation protocol for digital health investigators prior to full-scale deployment.

## Introduction

Shift-work schedules are indispensable for maintaining 24/7 patient care; however, their inherent irregularity can disrupt shift-working nurses’ circadian rhythms and compromise their ability to maintain healthy lifestyles [1]. Beyond schedule variability, the extreme physical and psychological demands of nursing serve as significant barriers to health-promoting behaviors, exacerbating stress and impairing sleep quality and dietary habits [1,2]. Given that nurse well-being is a critical determinant of patient care quality [3], systematic efforts to support their health practices are essential. However, capturing the day-to-day fluctuations in shift-work routines requires long-term, continuous assessment to accurately document the dynamic interplay between work-related stressors and health outcomes [4]. Although nurses’ work-life and health-related behaviors are commonly monitored for approximately 2 weeks using diary-based questionnaires [5], traditional paper-based diaries present challenges, including the inconvenience in data entry for nurses and difficulties in data handling for researchers. Overcoming these barriers is crucial for advancing research on nurses working under irregular shift schedules.

Mobile health (mHealth) apps have emerged as transformative tools in health research, extending beyond health management and clinical intervention to serve as practical tools for real-time, repeated data collection [6,7]. For nurses, whose work involves irregular schedules and high-intensity demands, mHealth technologies offer practical methodological advantages over conventional approaches. Specifically, these tools facilitate real-time, continuous monitoring of health behaviors and professional challenges, effectively overcoming the spatial and temporal constraints inherent in the nursing environment [4,8]. By allowing anytime, anywhere data entry, mHealth apps enhance participant convenience and engagement, which are important for supporting adherence and data completeness in longitudinal research.

The integration of mHealth into research methodology addresses the key limitations of conventional data collection, including recall bias and participant compliance. Unlike retrospective paper-based surveys, mobile platforms allow ecological momentary data capture in naturalistic settings, supporting flexible, time-sensitive assessment of daily experiences and behaviors [4,9]. Furthermore, the interactive nature of these apps allows for the incorporation of motivational strategies to sustain participant adherence, thereby improving data completeness [10]. While previous mHealth initiatives and ecological momentary assessment (EMA) studies across various occupational contexts—such as nursing and shift-work environments—have demonstrated the potential of digital data capture [11,12], existing apps and off-the-shelf software (eg, REDCap, m-Path, and Google Forms) present critical operational and ergonomic limitations. Traditional EMA platforms typically rely on fixed-time notification schedules that cannot accommodate the rotating shift patterns (day, evening, and night) characteristic of clinical nursing [13,14]. Capturing contemporaneous data from shift workers requires dynamic notification logic synchronized with shift transitions, paired with adaptive follow-up prompts to mitigate missing entries without inducing survey fatigue. Furthermore, standard digital forms often depend on text-heavy dropdown menus or linear structures that impose substantial cognitive load on clinicians experiencing high fatigue after demanding rotations [15]. Generic platforms also struggle to continuously integrate disparate daily metrics—such as sleep, meal timing, and work-recovery dynamics—within a single, time-stamped timeline. These specific operational constraints underscored the necessity of a bespoke mHealth app, designed to streamline user entry, enforce temporal integrity, and support near-real-time data capture in fast-paced hospital environments. However, successful implementation requires a comprehensive evaluation that bridges the gap between technological functionality and research utility [16]. A dual-perspective approach that incorporates both end-user experiences and expert researcher insights can ensure that the tool is both practical and methodologically rigorous [17]. Leveraging mHealth through this framework may provide a practical and methodologically rigorous system for capturing the complex, dynamic lifestyles of nurses and may contribute to a more nuanced understanding of nursing workforce well-being.

The Nurses’ Work-Life and Health project was designed to assess shift-work nurses’ daily work experiences and health practices over a 2-week period. Timely and repeated reporting is critical for reducing recall bias and improving data completeness in longitudinal research, underscoring the importance of a shift-tailored mobile platform. To guide this investigation, this study addressed the following primary research question: “Is a shift-tailored, bespoke mHealth app feasible, acceptable, and methodologically robust for collecting high-fidelity, near–real-time longitudinal data on shift-working nurses’ work-life and health practices without imposing excessive user burden?” To answer this question, the primary objective of this study was to propose and demonstrate a transferable, dual-perspective evaluation framework for mHealth data collection tools in high-burden occupational settings, using the newly developed “Nurses’ Work-Life and Health” app as a tailored exemplar. While alpha and beta testing were conducted to optimize the app’s usability and user interface (UI), the core evaluation integrated 3 complementary data streams: end-user feasibility metrics from shift-working nurses, objective server-generated usage logs, and expert qualitative assessment from a PhD-trained researcher with expertise in longitudinal methodologies. By establishing this multidimensional framework, this study aims not only to validate a bespoke mHealth tool for continuous data collection in nursing research but also to provide a replicable protocol for evaluating similar digital research tools in occupational and digital health research.

## Methods

### Study Design and Overall Framework

To establish and demonstrate a transferable, dual-perspective evaluation framework for mHealth research instruments in high-burden occupational settings, this study used an iterative design and development methodology coupled with multitiered usability and feasibility evaluations, using the newly developed “Nurses’ Work-Life and Health” mobile app as an empirical exemplar. The app was designed to collect daily work- and health-related data from shift-work nurses over a 14-day period. An iterative, user-centered design was adopted, consisting of 3 sequential phases: conceptual framework and needs assessment (phase 1); app design and development involving alpha testing (internal usability testing) with nurse researchers (n=5) to refine system logic and detect technical bugs, followed by beta testing (user-based usability evaluation) with practicing clinical nurses (n=16) to evaluate real-world UI/user experience (UX) ergonomics and standardized usability (phase 2); and a 14-day pilot feasibility study with nurse end users (n=5) as a methodological proof-of-concept, complemented by qualitative process evaluation with an expert nurse researcher (phase 3; Figure 1). This 3-phase structure was specifically aligned to evaluate the feasibility, adherence, temporal integrity, and user burden associated with app-based longitudinal data capture in clinical settings.

**Figure 1.**
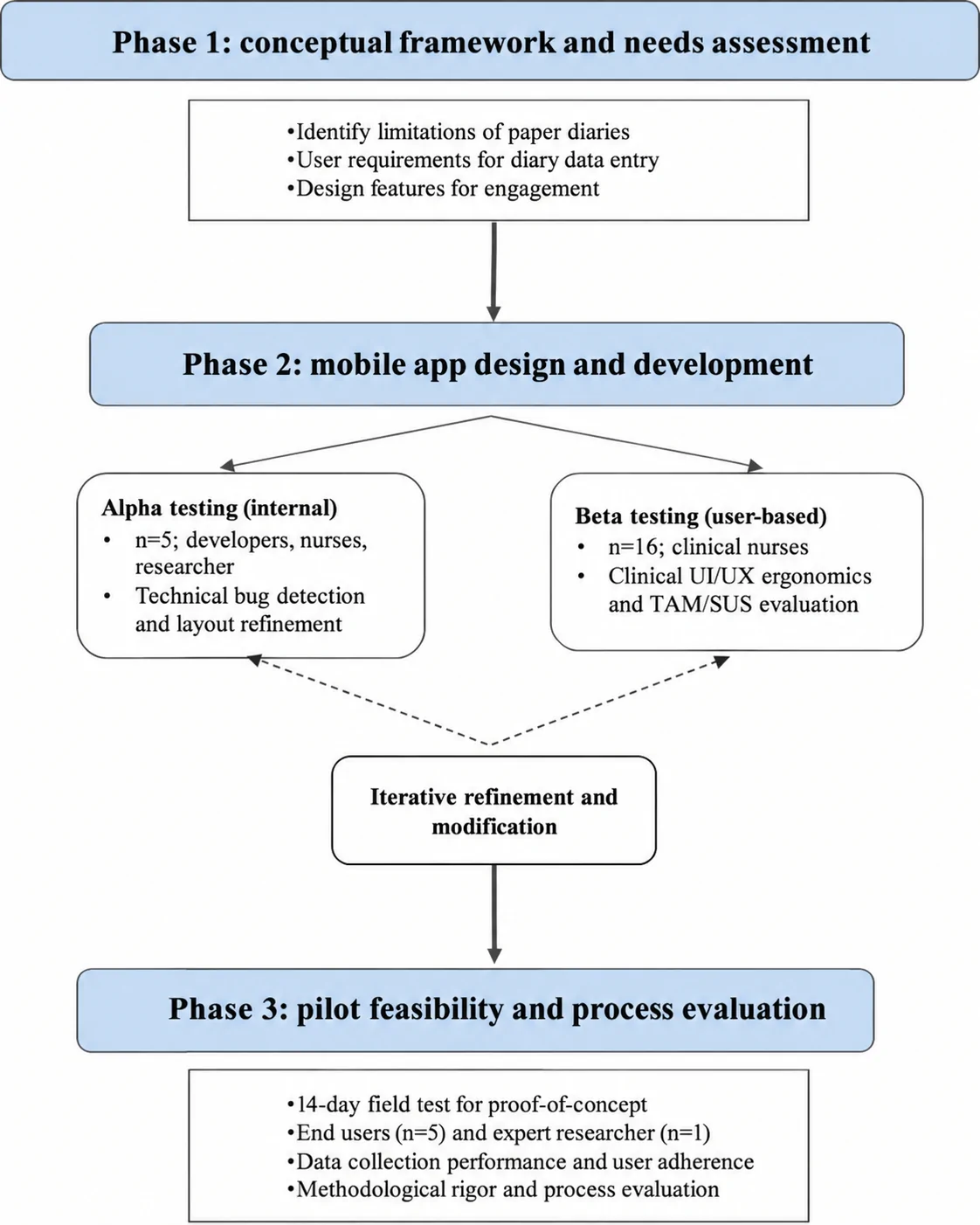
Study process. SUS: system usability scale; TAM: technology acceptance model; UI/UX: user interface/user experience.

### Phase 1: Conceptual Framework and Needs Assessment

#### Conceptual Framework

The conceptual framework underlying the app was informed by established models linking work characteristics and health, including the Job Demands–Resources model [18] and the Effort–Recovery model [19]. These models guided variable selection and questionnaire structuring, rather than being subjected to empirical testing or theoretical extension in this study. Within this operational structure, nurses’ daily work characteristics (eg, shift type, working hours, and perceived demands) were mapped as work demands, while sleep duration/quality, physical activity, dietary patterns, and hydration were operationalized as daily recovery mechanisms and health outcomes. Using these frameworks as structural foundations ensured that the daily journal comprehensively captured the essential temporal dimensions of nurse work-life and recovery dynamics.

To assess the daily work- and health-related experiences of shift-work nurses, the study used a structured reporting approach to capture work- and health-related information. The questionnaire comprised three components: (1) baseline survey (demographic and occupational background), (2) daily journal administered over 14 days (shift characteristics, sleep, nutrition/hydration, physical activity, and acute fatigue), and (3) postassessment survey (overall work-related fatigue, stress, chronotype, and health practices). To ensure methodological transparency and clarify the clinical relevance of these measures, Table 1 details the specific variables collected within the daily journal, their measurement frequency and timing, and their intended conceptual relationship to nurses’ occupational health outcomes (eg, mapping shift demands to acute fatigue and sleep disruption as a recovery indicator).

**Table 1.** Overview of daily measures, collection timing, and relevance to nurse occupational health.

Data domain	Specific variables collected	Measurement timing/frequency	Relevance to nurses’ occupational health and research objectives
Shift and work characteristics	Shift type (day/evening/night/off), actual working hours, overtime, and perceived shift workload/demands	End of shift or end of day	Quantifies objective and subjective daily job demands; identifies workload spikes that trigger acute fatigue
Sleep and circadian rhythm	Sleep onset/wake time, total sleep duration, subjective sleep quality, and night awakenings	Upon waking	Captures day-to-day sleep disruption caused by rotating shifts; evaluates primary physiological recovery mechanism
Dietary and hydration behaviors	Meal timing, meal skipping, caffeinated beverage intake, and daily water consumption	End of shift/continuous	Assesses lifestyle health practices affected by high workload; monitors nutritional deficits and dehydration under fatigue
Physical activity and rest	On-duty movement/walking duration, leisure physical activity, and structured rest breaks during shift	End of day/sync	Evaluates physical exertion vs active recovery; examines physical strain associated with clinical duties
Subjective well-being and fatigue	Daily perceived fatigue level, acute stress level, and overall health perception (Likert scales)	End of shift/end of day	Tracks real-time fluctuations in acute fatigue and occupational stress, serving as immediate indicators of nonrecovery

#### Needs Assessment

##### Needs Assessment Procedures and Participants

A comprehensive needs assessment was conducted to inform app design, incorporating perspectives from both clinical end users and researchers. For user-oriented needs assessment, semistructured discussions were held with 5 shift-work nurses to identify limitations of paper-based diary methods, practical requirements for daily data entry in clinical settings, and essential features for sustained engagement. Five shift-work nurses with at least 2 years of clinical experience and prior exposure to paper-based research diaries were recruited via purposive and snowball sampling to identify practical documentation barriers during clinical shifts. Discussions focused on practical barriers to documentation during high-fatigue shifts and user preferences for digital interfaces. In parallel, 2 senior nurse researchers specializing in occupational health and longitudinal methodologies were purposefully selected to define researcher-side functional requirements. This process aimed to identify critical functional requirements for high-quality data collection, focusing on data integrity: automated time-stamping to verify “near–real-time” entry and prevent retrospective “back-filling;” administrative efficiency: reducing the burden of manual data cleaning and entry associated with paper diaries; and real-time adherence monitoring: allowing timely intervention in cases of nonresponse to maintain protocol compliance.

The needs assessment was structured around several core conceptual domains, categorized into user-oriented and researcher-oriented requirements, ensuring a balanced design that met both clinical and methodological needs.

##### User-Oriented Requirements

The end-user perspective focused on 5 primary operational and functional specifications essential for sustainable longitudinal tracking in clinical shift-work environments:

1. Mitigation of recall bias via contemporaneous capture: addressing reported entry delays, the app required shift-synchronized dynamic notifications paired with automated server-side time-stamping to verify near–real-time data logging and eliminate retrospective recall bias.
2. Minimization of daily burden and survey fatigue: to accommodate demanding clinical workloads, questionnaire length and input structures were optimized for rapid, low-friction entry, ensuring daily completion within a few minutes without interfering with clinical duties.
3. Cognitive load reduction through item simplification: variable phrasing across work demands, sleep, nutrition, and recovery domains was streamlined and operationalized into concise, intuitive items to prevent cognitive overload during postshift fatigue.
4. Temporal alignment with work—recovery frameworks: item architectures were structured to capture day-level fluctuations across preshift, postshift, and recovery windows, ensuring direct alignment with occupational health frameworks (Job Demands–Resources and Effort–Recovery models).
5. Ergonomic, time-oriented interface design: UI specifications prioritized visual clarity and ease of navigation, incorporating a dynamic calendar layout, timeline-based progress tracking, and single-touch visual toggles over open text-entry fields.

##### Researcher-Oriented Requirements (Methodological Rigor)

In parallel, researchers’ requirements for longitudinal studies were identified:

1. Automated data management: to eliminate human error and the administrative burden associated with manual data entry from paper diaries, a secure back-end cloud infrastructure was required.
2. Real-time adherence monitoring: researchers identified the need to monitor user engagement in real time, allowing timely intervention for persistent nonresponse.
3. Temporal integrity and verification: time-stamped entries were essential to verify data collection within designated windows, thereby ensuring the methodological rigor of the data capture protocol and preventing retrospective “back-filling.”

### Integration of Needs Assessment Findings Into App Design

Insights from both user- and researcher-oriented assessments indicated that existing, off-the-shelf survey tools were insufficient for the constraints of shift-work nursing, directly shaping the specifications for a bespoke mHealth app. From the user perspective, key design priorities included shift-synchronized notification logic with adaptive follow-up prompts, concise questionnaires designed for completion within 3 to 4 minutes, and an ergonomic visual layout that minimizes cognitive burden during postshift fatigue. From the researcher perspective, key design priorities included a secure back-end infrastructure capable of generating time-stamped logs and a cloud-based dashboard for real-time monitoring of data completeness.

During initial planning, the app’s architecture was systematically mapped using a hierarchical tree structure to define relationships among disparate data domains (eg, shift timing, sleep metrics, and daily fatigue) and optimize screen navigation flows. This hierarchical organization ensured that complex multidomain entry forms were decomposed into intuitive, low-cognitive-burden visual modules before software engineering commenced. Preliminary screen layouts and core functionalities were outlined to ensure intuitive navigation and minimal user burden. A detailed development plan was created using Microsoft PowerPoint, specifying screen layouts, UI elements, and functional requirements. To enhance usability, selected design features from existing calendar-based apps were incorporated, allowing users to visualize daily entries relative to work schedules. Sample screens were developed using SketchUp (Trimble Inc) on an iPad to provide a visual prototype. Throughout development, contemporary literature on diary methods and mHealth interventions was consulted to ensure that the app was theoretically grounded and methodologically sound.

### Phase 2: Mobile App Design and Development

#### Development Team and Process

The “Nurses’ Work-Life and Health” mobile app was developed through interdisciplinary collaboration involving a university-affiliated supervisor with expertise in computer science and engineering and 2 mobile app developers. This collaborative framework supported alignment between technical development and research requirements throughout the iterative development process. Real-time collaboration via Discord facilitated continuous discussion of functional requirements and technical revisions.

#### Platform Selection and Technical Infrastructure

The app was developed for the Android operating system, chosen for its broad global market share, accessibility across diverse user populations, and open-source operating system, which allows flexible customization and integration. Google Firebase was used to support both front-end and back-end development. Cloud Firestore (Google LLC) was used for real-time data storage and synchronization, allowing real-time data transmission and secure storage of user-entered information. Google Firebase’s real-time database functionality also facilitated monitoring of data completeness and system performance during testing.

#### Data Security and Privacy

Data confidentiality and integrity were supported through the robust security infrastructure of Google Firebase. All data were encrypted at rest using the Advanced Encryption Standard (AES)-256 within Cloud Firestore and in transit via SSL/HTTPS/TLS protocols to prevent unauthorized interception. Participant authentication was managed using Firebase Authentication (Google LLC), which assigned unique, nonidentifiable user identifiers to decouple personal information from research data. Access control was enforced through Firebase Security Rules, restricting participants to their own records and providing researchers access only to deidentified datasets. These measures supported the secure handling of sensitive health information throughout the 14-day study period.

#### Real-Time Data Capture Protocol and Data Integrity Safeguards

To enforce contemporaneous data reporting and prevent retrospective “back-filling,” rigorous technical rules were implemented within the app’s architecture:

##### Notification Schedule and Adaptive Follow-Up Prompts

To prevent survey fatigue and minimize reporting burden during active duty, automated push notifications were dynamically scheduled based on each participant’s recorded shift type (day, evening, night, or off) rather than deploying fixed clock-time reminders. Specifically, once a participant configured their rotating shift pattern via the schedule interface, the app dynamically triggered postshift journal notifications aligned with shift termination windows: 2 PM for day shifts, 9 PM for evening shifts, and 8 AM (next morning) for night shifts, with off-duty prompts scheduled at 9 PM for daily summary reflection. If no data entry was recorded following the dynamic prompt, the back end executed an automated reengagement protocol, sending an adaptive follow-up reminder 2 hours post trigger to encourage timely completion within the permitted window.

##### Permitted Completion Windows and Time-Stamping Procedures

Each daily journal module was governed by a strict 4-hour permitted completion window starting from the scheduled notification time. To guarantee high-fidelity temporal tracking, every data entry was automatically embedded with a server-side UTC time stamp generated by Cloud Firestore upon database submission, completely independent of the user’s local device clock.

##### Handling of Delayed or Out-of-Window Entries

Entries submitted within the 4-hour window were classified as contemporaneous “near–real-time” data. If a participant attempted to log data beyond the 4-hour window, the app permitted data submission to preserve overall data completeness, but the back-end system automatically flagged these records as “delayed entries.” These flagged entries were isolated during data preprocessing, enabling researchers to verify protocol adherence objectively and exclude delayed reports from primary near–real-time sensitivity analyses.

### Alpha Testing

Alpha testing was conducted to evaluate the initial prototype’s stability, functional integrity, and visual ergonomics from a multidisciplinary perspective before clinical exposure. Consistent with Nielsen and Landauer’s [20] recommendation that testing with 5 users can identify approximately 85% of usability problems, 5 experts—including 2 mobile app developers, 2 nurse researchers, and 1 clinical nurse—were purposively recruited and participated in testing. In this phase, participants primarily functioned as technical and structural evaluators tasked with identifying system bugs, workflow errors, and UI/UX layout inconsistencies.

A mixed methods approach was adopted incorporating a quantitative survey instrument adapted from the technology acceptance model [21] and the system usability scale [22] to systematically assess technical functionality and user-centered design. Quantitatively, participants rated 17 items across six domains using a 4-point Likert scale (1=“strongly disagree” and 4=“strongly agree”): (1) ease of use (eg, navigation of the calendar and timeline), (2) functionality (eg, data modification and information display), (3) user satisfaction, (4) technical performance, (5) perceived usefulness, and (6) intention to use. The 4-point scale was specifically selected to avoid neutral responses and elicit more definitive feedback. Qualitative data collection integrated domain-specific open-ended questionnaire items (eg, “If you encountered any issues or gave a low rating, please describe the experience in detail”), providing granular qualitative feedback on technical bugs, system crashes, or navigation barriers. The combined findings directly informed back-end system revisions and UI refinements prior to beta testing.

### Beta Testing

Following alpha testing refinements, beta testing was conducted to evaluate usability and feasibility in a real-world clinical context with 16 clinical shift-work nurses across diverse hospital units (eg, medical-surgical ward, intensive care unit [ICU], and emergency department [ED]). Participants were recruited via snowball sampling. Inclusion criteria for nurse participants required being a registered nurse with at least 6 months of clinical nursing experience, working a rotating shift schedule, and owning an Android-based smartphone. Nurses on extended medical or maternity leave, those working fixed day/night schedules, or those assigned exclusively to nonclinical administrative units were excluded. The focus was on the integration of the app into the demanding and irregular workflows of nursing professionals.

Similar to alpha testing, a mixed methods approach was used to evaluate clinical usability and UX in real-world nursing environments. Quantitative data collection used a structured survey instrument adapted from the technology acceptance model [21] and the system usability scale [22]. Participants completed a 15-item survey across six domains: (1) ease of use (eg, intuitive data entry during high-fatigue states), (2) functionality (eg, flexibility of data modification and app environment stability), (3) user satisfaction (eg, visual legibility of icons and colors), (4) technical performance (eg, app loading speed), (5) perceived usefulness (eg, effectiveness in monitoring the relationship between shift-work and health behaviors), and (6) user burden and intention to use (eg, minimal interference with clinical duties and willingness for 14-day continuous use). All items were rated on a 4-point Likert scale (1=“strongly disagree” and 4=“strongly agree”).

To capture contextual nuances specifically regarding operational barriers to app use, qualitative data collection integrated domain-specific open-ended survey questions (eg, “If you encountered difficulties during a specific shift, please describe the circumstances in detail”). This allowed researchers to identify how specific clinical circumstances and temporal constraints (such as high-intensity patient handovers or emergency admissions) directly impeded app interaction and timely data entry. Addressing these user-reported difficulties, qualitative feedback directly guided functional app refinements, such as extending permitted completion windows and optimizing dynamic push notification timing, thereby minimizing user friction and potential participant attrition prior to the 14-day feasibility study.

Through these iterative alpha and beta testing cycles, the UI and UX were optimized for clinical shift-work environments (Figure 2). Specifically, in addition to the main dashboard displaying daily summary metrics and timeline status (Figure 2A), the shift schedule configuration interface was designed to allow nurses to seamlessly set their rotating shift patterns (day, evening, night, off, or others), which automatically synchronizes dynamic postshift notification triggers (Figure 2B). Furthermore, to minimize cognitive friction during postshift fatigue, the daily recovery and health journal interface was refined using high-legibility visual sliders (eg, a 1‐10 scale for measuring sleep quality, subjective health, and daily stress levels) and streamlined inputs, enabling rapid and intuitive data capture without text-entry burden (Figure 2C).

**Figure 2.**
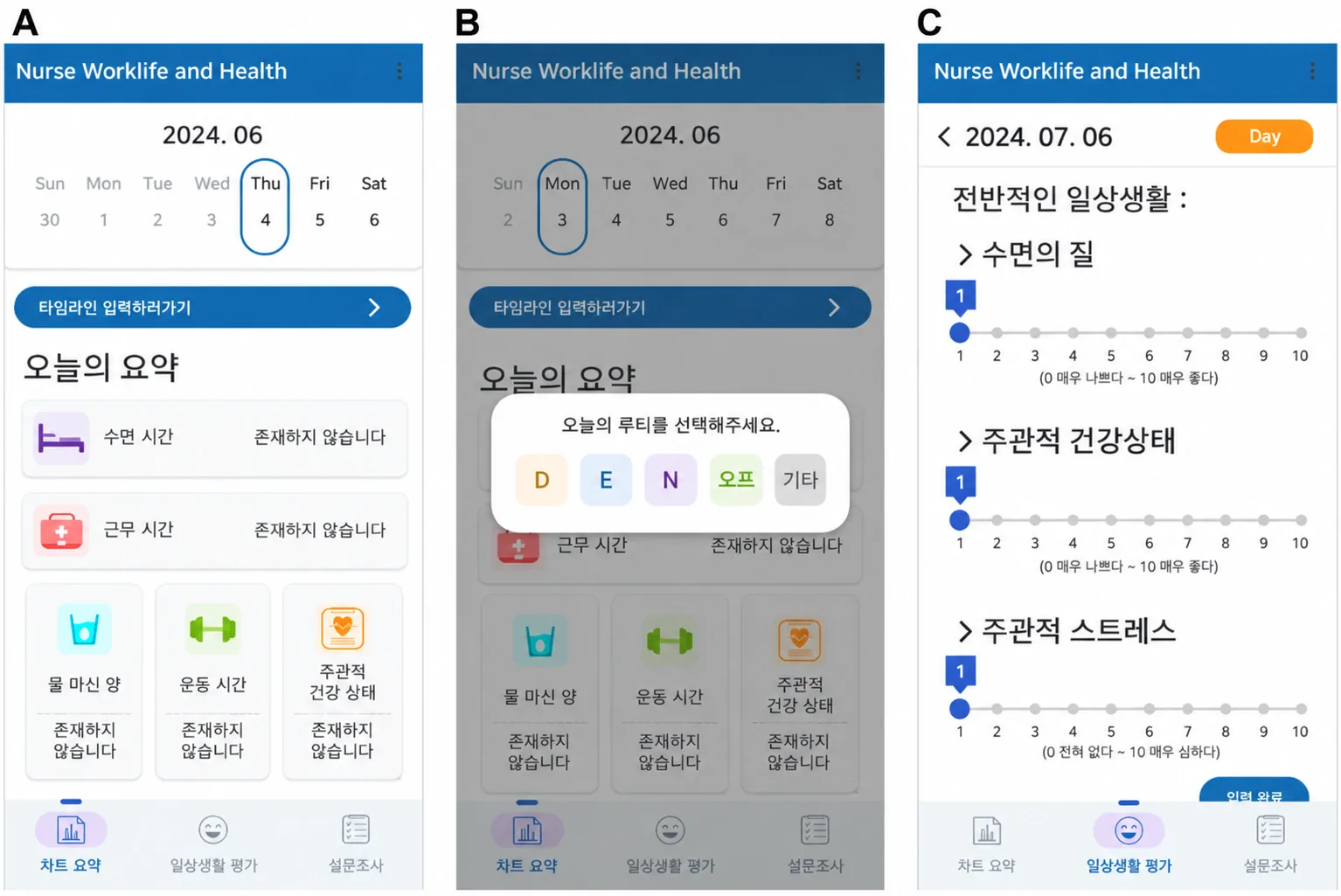
Screenshots of Nurses’ Work-Life and Health app. (A) Main dashboard displaying daily summary metrics and timeline status. (B) Shift schedule configuration pop-up, allowing users to select rotating shift patterns (day, evening, night, off, and others), which automatically syncs postshift dynamic notification triggers. (C) Daily health and recovery input interface, demonstrating the 1 to 10 visual sliders for measuring sleep quality, subjective health status, and stress levels with minimal cognitive load.

### Phase 3: Feasibility and Process Evaluation

#### Feasibility and Process Evaluation Overview

The final phase consisted of a 14-day feasibility and process evaluation to assess the app’s performance, sustainability, and methodological rigor in real-world settings. This phase comprised 2 complementary components: an end-user feasibility study involving 5 shift-work nurses and an expert process evaluation with a PhD-trained nurse researcher experienced in longitudinal research design and data analysis. All 6 participants engaged in daily data collection via the app for the full 14-day period to ensure firsthand evaluation.

For the end-user component, the practical utility and methodological proof-of-concept of the mHealth tool were evaluated across five domains: (1) adherence, (2) completion, (3) user burden, (4) acceptability, and (5) technical issues. To enhance methodological rigor, objective system logs were triangulated with subjective postuse surveys. Adherence was operationalized as the proportion of active study days during which participants logged into the app and recorded data, calculated using a total denominator of 70 participant-days (5 participants×14 consecutive monitoring days). Completion was defined as the proportion of actual journal entries submitted relative to the 70 expected daily journal entries. Adherence, completion, and server-side submission time stamps were automatically recorded by Cloud Firestore to establish objective protocol tracking without self-report bias. Similar to phase 2 beta testing, 5 shift-work clinical nurses were recruited via snowball sampling, targeting registered nurses with at least 6 months of clinical experience working rotating shifts on Android smartphones. To ensure diversity in high-burden clinical workflows, recruited participants represented distinct specialty units, including general medical-surgical wards, ICU, and ED. Quantitatively, data collection used a poststudy feasibility questionnaire instrument adapted to evaluate user burden (perceived interference with clinical duties), acceptability (overall satisfaction and willingness for sustained 14-day tracking), and technical stability (absence of system crashes or sync failures) using a 4-point Likert scale.

To gain deeper methodological insights into the data collection process, an in-depth qualitative process evaluation was conducted with an expert nurse researcher who actively used the app daily throughout the 14-day study period. Purposive sampling was used to recruit an expert who possessed dual expertise as both a practicing clinical nurse and a PhD-trained nurse researcher with extensive experience in longitudinal study design, mHealth methodologies, and occupational health data analysis. Qualitative data collection used a semistructured interview instrument comprising open-ended questions targeting four predefined operational domains: (1) operational usability during clinical shifts, (2) perceived data accuracy and recall reduction, (3) potential measurement biases (eg, behavioral reactivity), and (4) recommendations for technical scalability. The individual interview was conducted online by a faculty researcher (KH) with doctoral-level expertise in qualitative methodologies, lasting approximately 40 to 50 minutes via a secure video-conferencing platform (Zoom) following the 14-day data collection period. The session was audio-recorded with prior written informed consent and transcribed verbatim, with supplementary written responses obtained for member-checking. Qualitative data were analyzed using a qualitative descriptive approach combining deductive framework analysis and inductive thematic coding. Initial coding was deductively structured around four predefined primary themes derived from our evaluation framework: (1) self-awareness and behavioral reflection, (2) strengths of the app as a research tool, (3) limitations and potential measurement biases, and (4) recommendations for expansion and refinement. Within these primary domains, secondary subthemes were inductively generated from the transcript data. To ensure analytical rigor and trustworthiness, 2 researchers (KP and KH) independently coded the transcript, met iteratively to resolve discrepancies and refine code definitions, and triangulated qualitative feedback with objective system-use logs. Finalized themes were reviewed with the participant (member checking) to confirm representativeness.

#### Iterative Refinement

Findings from alpha and beta testing and the final feasibility/process evaluation were integrated into successive versions of the app through an iterative refinement process. User feedback informed improvements in navigation, interface design, system stability, and data entry workflows. This iterative approach ensured that the final version of the “Nurses’ Work-Life and Health” mobile app aligned with both research objectives and user needs

### Ethical Considerations

The study protocol was reviewed and approved by the institutional review board of Chung-Ang University (approval number 1041078‐20240131-HR-023) prior to participant recruitment and data collection. Formal written informed consent was obtained from all individual participants prior to enrollment in each phase of the study. The consent process outlined the study objectives, data collection procedures, confidentiality safeguards, and the right to withdraw at any time without any professional or administrative penalty. To protect participant privacy and maintain confidentiality, all electronic research data were strictly deidentified. Upon registration, the back-end authentication system automatically assigned a nonidentifiable user identifier to decouple research datasets from personal identity information. Data transmissions were encrypted in transit via SSL/HTTPS protocols, and all stored records within Cloud Firestore were encrypted at rest using the AES-256 standard, with administrative access restricted strictly to primary investigators. To acknowledge their time and effort, clinical nurses who completed the 14-day feasibility protocol received a mobile electronic gift voucher valued at approximately US $35 (50,000 KRW), while participants in the alpha and beta testing phases received nominal token compensation (coffee coupons valued at US $5‐10) upon survey completion.

## Results

### Alpha Testing

#### Quantitative Usability Outcomes

Alpha testing demonstrated high overall usability and acceptance, with a total mean score of 3.47 (SD 0.53; Table 2). The highest ratings were observed for perceived usefulness (mean 3.80, SD 0.45) and intention to use (mean 3.80, SD 0.45), indicating strong alignment with research objectives and nursing practice needs. Favorable ratings were also reported for functionality (mean 3.40, SD 0.55). However, lower scores were observed for technical performance (mean 3.00, SD 0.71) and ease of use (mean 3.30, SD 0.55), reflecting challenges in timeline-based activity entry, calendar navigation, and initial intuitiveness for users without prior training.

**Table 2.** Summary of usability and acceptance ratings across alpha (n=5) and beta (n=16) testing phases.

Domain	Key assessment focus			
	Alpha test (n=5)	Mean (SD)	Beta test (n=16)	Mean (SD)
Ease of use	Navigating calendar screen, entering activities in timeline, and intuitiveness without training	3.30 (0.55)	Navigating and entering data during shift hours or in busy states	3.44 (0.63)
Functionality	Modifying entered data, clarity of displayed information, and data synchronization	3.40 (0.55)	Flexibility of data modification and app stability	3.31 (0.48)
User satisfaction	UI^a^ design satisfaction, preference over paper diaries, and overall experience.	3.20 (0.45)	Visual legibility (icons and colors) and overall preference over paper-based diaries	3.56 (0.51)
Technical performance	System speed, absence of crashes/bugs, and offline data handling	3.00 (0.71)	App loading speed and absence of crashes during rapid task switching	3.19 (0.75)
Perceived usefulness	Accuracy of health tracking, efficiency in shift-work monitoring, and data value	3.80 (0.45)	Value of monitoring the relationship between shift-work and health behaviors	3.75 (0.45)
Intention to use	Intent for 14-day continuous use and willingness to recommend to colleagues	3.80 (0.45)	Minimal interference with clinical duties and intent for 14-day continuous use	3.63 (0.50)
Total average		3.47 (0.53)		3.48 (0.55)

a UI: user interface.

#### Qualitative Feedback and Functional Refinements

Open-ended responses provided actionable insights for addressing lower-rated domains, which were categorized into three primary themes:

1. Navigation and ease of use: participants reported initial difficulties during registration and suggested that timeline activity selection should be more visually prominent. To resolve this, a structured onboarding tutorial was implemented, and the activity selection UI was redesigned with higher contrast and clearer labeling to improve intuitiveness for first-time users.
2. Functionality and technical performance: participants emphasized the need for a more seamless data modification process and identified intermittent bugs related to status indicators. Accordingly, the data modification workflow was streamlined to reduce the number of steps required, and real-time progress icons were added to allow users to monitor survey completion efficiently.
3. Visual design for usability: although the overall layout received positive comments, experts recommended stronger visual differentiation to support high-fatigue users. To reduce cognitive load, a comprehensive color-coding system and icon set were incorporated, categorizing activities (eg, work, sleep, and meals) into distinct visual groups.

#### Modifications Following Alpha Testing

Based on the synthesis of quantitative and qualitative findings, final revisions focused on improving navigation clarity, streamlining diary entry workflows, and enhancing system stability. These refinements ensured that the app could effectively minimize user burden and capture high-quality, real-time data during the subsequent 14-day feasibility study.

### Beta Testing

#### Quantitative Usability Outcomes

Beta testing (n=16) supported the app’s usability in a real-world clinical context, with a total mean score of 3.48 (SD 0.55; Table 2). The highest ratings were observed for perceived usefulness (mean 3.75, SD 0.45) and intention to use (mean 3.63, SD 0.50), suggesting strong acceptance by clinical nurses. User satisfaction was also favorable (mean 3.56, SD 0.51). In contrast, technical performance received a relatively lower score (mean 3.19, SD 0.75), indicating a need for further optimization of system stability and loading speed in fast-paced clinical settings.

#### Qualitative Feedback and Contextual Insights

Qualitative analysis of open-ended responses identified three primary themes related to the practical use of the app.

1. Navigation and ergonomics: nurses initially found calendar navigation unintuitive. Specifically, button size and placement hindered rapid data entry between shifts, highlighting the need for a “thumb-friendly” design for on-the-go use.
2. Data visualization and summarization: participants appreciated the timeline-based visualization but requested enhanced summary features, such as weekly health overviews, to better track long-term patterns. Color-coding and icon-based differentiation were emphasized to improve readability.
3. System stability and engagement: intermittent technical issues, such as crashes when multitasking, were reported. Participants suggested integrating interactive features, including automated health tips and push notification reminders, to sustain engagement.

#### Modifications Following Beta Testing

Based on the synthesized results, final modifications were made to stabilize the system and optimize the UI. Key revisions included (1) ergonomic UI adjustments (button sizes and navigation flows were recalibrated to facilitate one-handed use during busy clinical hours), (2) enhanced visual hierarchy (icon sets and color schemes were finalized to clearly differentiate work, sleep, and health activities, minimizing cognitive load), and (3) stability and notification logic (system architecture was optimized to prevent crashes during rapid multitasking, and a personalized push notification system was integrated to maintain adherence).

### Feasibility Testing

The 14-day feasibility evaluation demonstrated strong protocol feasibility and high temporal fidelity. Across the 70 total participant-days (5 participants × 14 d), objective adherence reached 94.3% (66/70 participant-days; mean 13.2, SD 1.1 d/nurse) with 100% participant retention (Table 3). Of the 70 expected daily journal entries, participants successfully completed 62 entries, yielding an overall completion rate of 88.5% (62/70). The analysis of server-side UTC time stamps confirmed that 83.9% (52/62) of completed entries were submitted within the strict 4-hour completion window following dynamic shift-matched notifications, supporting the feasibility of near–real-time data capture in fast-paced clinical environments. The remaining 16.1% (10/62) of entries were submitted beyond the 4-hour window and flagged by the back end as delayed entries, reflecting periods of acute workload surges or extended clinical handovers. System logs showed that the average time spent per entry was 3.4 (SD 0.8) minutes, which was rated as highly acceptable (mean 3.70, SD 0.50) and associated with low perceived interference with daily routines (mean 3.65, SD 0.45), despite demanding shift schedules.

**Table 3.** Summary of feasibility test outcomes (n=5).

Category and indicator/item	Outcome
Adherence	
Participant retention rate (14 d), %	100.0
Objective protocol adherence rate (participant-days), n/N (%)	66/70 (94.3)
Mean days of app use (out of 14 days), mean (SD)	13.2 (1.1)
Completion	
Overall journal completion rate (expected entries), n/N (%)	62/70 (88.5)
Entries within 4-hour window (near–real-time) (completed entries), n/N (%)	52/62 (83.9)
Delayed entries (>4 h postprompt) (completed entries), n/N (%)	10/62 (16.1)
Mean time per entry (min), mean (SD)	3.4 (0.8)
User burden	
Low perceived interference with daily routines, mean (SD)	3.65 (0.45)
Acceptability of entry frequency, mean (SD)	3.70 (0.50)
Acceptability	
Overall user satisfaction, mean (SD)	3.82 (0.38)
Willingness for long-term use, mean (SD)	3.68 (0.48)
Technical issues	
System stability and absence of critical bugs, mean (SD)	3.45 (0.65)
Ease of troubleshooting (if any), mean (SD)	3.50 (0.70)

A semistructured interview with the participating expert nurse researcher, who used the app daily over the 14-day period, highlighted several points:

1. Self-awareness and behavior reflection: daily logging increased the researcher’s awareness of personal health patterns, including irregular sleep, suboptimal hydration, and habitual early awakening. The researcher reported voluntary attempts to improve these behaviors, suggesting the potential for the app to function as a behavioral intervention tool.
2. Strengths as a research tool: the researcher described the interface as intuitive and highly appropriate for real-time or near–real-time data capture. In comparison to paper-based diaries or static digital forms (eg, Google Forms), the app was perceived as significantly more convenient, reducing recall bias and facilitating streamlined data review and management.
3. Limitations and potential biases: critical methodological considerations were identified, including reliance on self-reported data, potential behavioral reactivity (Hawthorne effect), and practical challenges of entering data during peak clinical workload. Technical limitations included occasional survey resets and absence of built-in validation for implausible entries, which may affect data integrity.
4. Recommendations for expansion and refinement: the researcher suggested strengthening item linkage and workflow prompts to minimize respondent burden, refining contextual item structures (eg, meal location), and separating patient safety incident categories to improve interpretability. For large-scale implementation, the researcher recommended incorporating diverse data fields and robust automated data-quality management systems.

Overall, the 14-day feasibility and process evaluation suggested that the app has practical value for both research data collection and self-monitoring, while also identifying opportunities for further refinement in technical performance and workflow design.

## Discussion

### Principal Results and Evaluation Framework Implications

This study developed and evaluated the “Nurses’ Work-Life and Health” app to address the methodological challenges of longitudinal data collection in shift-working nursing populations. Beyond demonstrating the technical viability of the app, the principal scientific contribution of this study lies in its transferable, dual-perspective evaluation framework. By integrating subjective end-user experiences, objective back-end system logs, and in-depth qualitative scrutiny from an expert researcher who directly engaged in the protocol, this framework bridges the critical gap between technological functionality and research utility. When interpreting these outcomes, it is essential to distinguish between context-specific pilot findings and the broadly transferable aspects of our methodology. Given the pilot scale of the feasibility evaluation (n=5), specific quantitative indicators—such as the 100% retention rate, 94.3% adherence rate, and 3.4-minute entry time—represent context-specific feasibility metrics bounded by this particular clinical sample. In contrast, the broader scientific contribution resides in the transferable design mechanisms and methodological principles established through this work. Specifically, integrating shift-synchronized notification logic with low-cognitive timeline interfaces demonstrates how mHealth tools can minimize reporting burden in high-fatigue settings, while the 3-tiered evaluation protocol offers a replicable strategy for digital health investigators seeking to validate data collection tools in complex occupational environments.

The high level of engagement achieved in this study may be primarily attributed to the app’s ease of use and efficiency. Although initial qualitative feedback from alpha and beta testing highlighted challenges with navigation for high-fatigue users, iterative refinements—including ergonomic “thumb-friendly” adjustments and enhanced visual differentiation—appeared to mitigate these concerns. Objective system logs showed that the average time required for each entry was 3.4 minutes, suggesting minimal time burden for repeated reporting. This low entry burden is meaningful in the context of repeated mobile diary reporting and EMA research, where participant compliance and sustained engagement are closely related to the practical burden of data entry [4,23]. The expert nurse researcher further corroborated these findings, noting that recall bias was significantly reduced by the app compared to paper diaries by allowing near–real-time data capture. By reducing the effort required for data entry, the app may reduce the back-filling of data common in retrospective reports, thereby enhancing the ecological validity of the longitudinal data [24].

Beyond its role as a data collection instrument, the app demonstrated potential as a self-monitoring tool. The participating expert nurse researcher reported increased self-awareness regarding health behaviors, such as hydration and sleep patterns. Although this behavioral reactivity could introduce bias in observational studies [25], it also highlights the app’s potential for self-monitoring and behavior reflection. While this study used the Job Demands–Resources and Effort–Recovery models as instrumental guides for variable selection, the high completion rates across daily work and recovery domains support the practical utility of these frameworks for structuring longitudinal mHealth diaries. Moving forward, future iterations could incorporate personalized feedback dashboards or real-time alerts, extending the app from a passive data collection tool into a closed-loop intervention platform [26,27]. Integration with wearable sensors could validate subjective diary data against objective physiological metrics (eg, heart rate variability) to provide a more nuanced understanding of the work-recovery process.

However, the expert process evaluation also emphasized the importance of methodological safeguards for broader implementation. Key concerns included delayed data entry during high-intensity shifts and the risk of satisficing (choosing the easiest response) under extreme fatigue [28]. To mitigate these risks and support data integrity, the app could incorporate smart-validation mechanisms and dynamic skip logic to filter irrelevant items and prevent inconsistencies in self-reported data [29]. The integration of context-aware prompts (eg, shift-synchronized notifications or GPS-based reminders) could ensure timely, accurate entries without disrupting clinical duties. Additionally, expanding the tool to include more diverse contextual variables (eg, specific patient safety incidents or unit-level staffing) would increase its utility for broader epidemiological or organizational research. By evolving into a more adaptive longitudinal data collection platform, the app can support the synthesis of high-fidelity evidence critical for nursing research.

The evaluation framework established in this study offers broader methodological utility for digital health research. In mHealth and EMA research, tools are frequently deployed without rigorous prevalidation of their impact on participant burden or data integrity [16]. Our 3-tiered evaluation strategy—combining iterative usability testing, objective compliance logging, and firsthand expert process evaluation—provides a standardized, replicable roadmap for researchers developing digital tools for other high-stress, shift-work, or clinical populations (eg, physicians and emergency responders). Adopting this dual-perspective assessment ensures that digital data collection instruments are not only technologically robust and user-friendly but also methodologically capable of minimizing recall and reporting biases.

### Limitations and Future Directions

Several limitations should be acknowledged. First, the app was developed exclusively for the Android platform, which may initially limit accessibility. However, this platform-specific focus facilitated the stabilization of the back-end architecture before cross-platform expansion. Second, while the sample sizes for usability testing (n=16) and feasibility evaluation (n=6) align with established usability engineering standards and pilot study guidelines [20,30], the quantitative implementation outcomes reported here are inherently preliminary and context-specific. Furthermore, the reliance on nonprobability sampling methods (purposive and snowball sampling) across testing phases introduces potential selection bias. Participants in this pilot may represent early adopters with higher digital literacy, greater intrinsic health awareness, or higher research engagement, which could overestimate adherence and satisfaction compared to broader clinical populations. In addition, while participants represented major high-burden units (medical-surgical wards, ICU, and ED), certain specialty areas—such as outpatient clinics or psychiatric units—were not covered, limiting the applicability of findings across all clinical contexts. Finally, while the title and conceptual framework emphasize nurses’ health, this 14-day pilot study primarily evaluated the feasibility, user acceptability, and temporal fidelity of the data collection platform rather than directly assessing long-term clinical health outcomes. Although the daily journal captures key health-related behaviors and recovery metrics, the lack of direct evaluation of health outcome trajectories represents a main limitation of this feasibility study. Accordingly, our conclusions are bounded by these pilot data, and the specific metrics should not be directly generalized as definitive population-level estimates. Rather, the primary value of this pilot lies in demonstrating the proof-of-concept for the dual-perspective evaluation framework and identifying structural refinements required for scalability. Future research must deploy this tool across larger, more heterogeneous nursing cohorts using probabilistic sampling and extended follow-up windows to evaluate long-term durability and generalizable implementation outcomes. To build upon this platform, subsequent iterations should also incorporate daily self-reported journal entries with objective physiological markers—such as wearable actigraphy or heart rate variability—to empirically evaluate the direct impact of shift work on nurses’ long-term clinical health outcomes.

### Conclusions

This study establishes and demonstrates a transferable, dual-perspective evaluation framework for assessing mHealth data collection tools in high-burden, shift-work occupational settings. By combining subjective end-user feasibility metrics, objective back-end usage logs, and qualitative process evaluation by an expert researcher, this multitiered approach bridges the gap between technical usability and methodological rigor. Applied to the newly developed “Nurses’ Work-Life and Health” app, the framework supported the feasibility of high temporal fidelity longitudinal data capture in complex clinical environments without imposing prohibitive cognitive burden on participants, as evidenced by high adherence (94.3%) and a brief 3.4-minute entry time.

Beyond the immediate evaluation of this specific app, this integrated evaluation strategy provides a replicable protocol for digital health investigators seeking to validate mHealth and EMA tools prior to full-scale deployment. While the framework demonstrated the app’s readiness for real-time monitoring, it also effectively identified crucial safeguards needed for future scalability, including dynamic skip logic, context-aware reminders, and automated data validation. Ultimately, this evaluation framework serves as a robust methodological foundation for future mHealth research, facilitating the collection of high-quality evidence needed to design data-driven interventions and policies that support nurses’ well-being and patients’ safety.

## Abbreviations

AES: Advanced Encryption Standard
ED: emergency department
EMA: ecological momentary assessment
ICU: intensive care unit
mHealth: mobile health
UI/UX: user interface/user experience
